# Achievement Motivation, Meaning in Life, and Well-Being Among Video Game Players

**DOI:** 10.3390/brainsci16010086

**Published:** 2026-01-12

**Authors:** Maciej Wierzbicki, Wojciech Rodzeń

**Affiliations:** Faculty of Social Sciences, Institute of Psychology, University of Szczecin, 70-453 Szczecin, Poland; 237483@stud.usz.edu.pl

**Keywords:** players, motivation, approach, avoidance, meaning in life, well-being

## Abstract

Background/Objectives: The present study aimed to examine the associations among achievement motivation, meaning in life, and well-being among video game players and to investigate differences between players with approach- and avoidance-oriented motivations. Methods: The sample consisted of 296 university students who reported playing video games (192 men and 104 women), aged 18 to 35 years (*M* = 22.62; *SD* = 2.64). Participants completed a battery of self-report measures, including the Achievement Goal Questionnaire, the Meaning in Life Questionnaire, and the WHO-5 Well-Being Index, administered anonymously. Results: Mediation analyses revealed that meaning in life was a significant mediator in the relationship between approach-oriented mastery goals and well-being (*Ind* = 0.07; 95% CI [0.02, 0.12]). However, no significant mediation effect was found for approach-oriented performance goals (*Ind* = 0.04; 95% CI [−0.01, 0.09]). Independent-samples *t*-tests indicated that participants with approach-oriented motivations reported significantly higher levels of meaning in life (*t*(294) = 4.44; *p* < 0.001), presence of meaning (*t*(294) = 5.74; *p* < 0.001), and well-being (*t*(294) = 5.52; *p* < 0.001) compared to those with avoidance-oriented motivations. Conclusions: The findings suggest that approach-oriented achievement motivations among players are positively associated with meaning in life and are indirectly associated with higher well-being, whereas avoidance-oriented motivations are associated with lower levels of well-being. These results carry potential implications for game design, education, and psychotherapy.

## 1. Introduction

Playing video games has become an increasingly common form of leisure activity [1]. Previous research has identified both positive and negative aspects of this pastime. Among the negative outcomes, researchers point to cultural misalignment, overstimulation, increased use of nicotine, and engagement in risky behaviors [2,3]. On the other hand, positive outcomes include enhanced cognitive abilities, creativity, and originality [4,5,6]. Further studies suggest that video games can be useful in education, the development of prosocial attitudes and communication skills [7], as well as in group therapy with neurodiverse adolescents—particularly those diagnosed with autism spectrum disorder or ADHD [8].

These aspects of gaming may foster personality development in multiple directions and help individuals discover meaning in life by providing opportunities to experience situations unavailable in real life, yet capable of forming cognitive representations in the mind [9,10,11]. Furthermore, some types of gaming have evolved into “e-sports,” defined as “playing games in which participants are oriented toward competition with one another—that is, any activity consisting of playing video games to defeat another player” [12] (pp. 214–215).

Video games may thus be approached from the perspective of competition, and consequently, achievement motivation [13]. Performance in games may be related not only to skills acquired through gameplay but also to players’ motivation and well-being, a notion supported by existing findings [14,15,16]. Moreover, video games constitute a unique ecological context for studying achievement motivation, as they are systems inherently designed around competence and progression. Unlike many real-world scenarios, games provide clear goals, immediate feedback loops, and explicit indicators of success such as levels, rankings, and achievements [17,18]. This specific structure makes the pursuit of mastery and performance metrics highly salient, thereby offering an optimal setting for examining how achievement motivation operates in practice [19].

The concept of achievement motivation was first articulated by Atkinson [20], who described it as an individual’s tendency to engage in an activity within two opposing orientations: the pursuit of success and the avoidance of failure. Based on Atkinson’s framework, three distinct motives can be identified: a positive achievement motive, referring to striving for success and goal attainment; a negative achievement motive, manifested in the avoidance of failure and its negative consequences; and an external achievement motive, resulting from environmental influences such as praise or criticism [21].

Other authors have emphasized that the foundation of achievement motivation research lies in understanding the causes of goal-directed activity [22]. Dweck distinguished between two types of achievement goals: those focused on learning and expanding one’s competencies, and those concerned with performance and the evaluation of already acquired competencies. Empirical studies [23] suggest that achievement goals are associated with behavior more directly than achievement motives. Building on this line of research, Elliot and McGregor [24] proposed a four-factor model of achievement goals, distinguishing mastery-approach, mastery-avoidance, performance-approach, and performance-avoidance orientations.

Achievement striving extends beyond performance improvement or reward acquisition—it also functions as a pathway to constructing meaning and purpose in one’s life. The pursuit of goals, whether in real or virtual contexts, may reinforce individuals’ belief in the value of their actions, thereby linking achievement motivation to the experience of meaning in life.

Steger et al. [25] proposed a definition of meaning in life that allows individuals to apply their own criteria to understand this construct. They defined meaning in life as “the sense made of, and significance felt regarding, the nature of one’s being and existence” [25] (p. 81). Based on their research, Steger and colleagues identified two dimensions of meaning in life: the presence of meaning and the search for meaning. Importantly, these dimensions are not necessarily correlated; thus, it is possible to search for meaning despite already perceiving life as meaningful, or conversely, to refrain from searching even in the absence of such a perception. Moreover, the presence of meaning has been found to correlate more strongly with well-being than the search for meaning [26].

Recent findings suggest that the presence of meaning in life may buffer depressive symptoms and reduce suicidal tendencies [27]. Research by Huda and colleagues [28] further demonstrates a differential relationship between life satisfaction and the two dimensions of meaning: life satisfaction is positively associated with the presence of meaning, whereas the search for meaning correlates negatively. Possessing a sense of meaning thus plays an important protective and motivational role, providing direction for daily activities and supporting the experience of positive emotions. Additional studies confirm that individuals with a strong sense of meaning in life are more likely to experience psychological well-being and to remain resilient in the face of adverse experiences [29,30,31]. Meaning in life can therefore be conceptualized as a mediating factor linking achievement motivation to well-being.

One of the widely cited definitions of well-being was proposed by Michaelson et al. [32], who described it as “how people feel and how they function both on a personal and social level, and how they evaluate their lives as a whole” (p. 6). Well-being has also been defined as an individual’s psychophysical state, subjectively experienced as positive [33]. Some scholars have noted the lack of sufficiently precise definitions of well-being, as well as significant discrepancies between its scientific and lay interpretations [34].

The concept may be approached through the Aristotelian lens of hedonism, understood as the pursuit of pleasure, and eudaimonism, defined as striving to live a valuable, virtuous, and authentic life [35,36]. Ryff [37] proposed a six-dimensional model of psychological well-being, comprising self-acceptance, positive relations with others, autonomy, environmental mastery, purpose in life, and personal growth. Contemporary studies continue to support the relevance of these dimensions, particularly positive relationships and a sense of purpose in life [38,39,40].

A study conducted among middle school students [41] demonstrated a link between achievement motivation—specifically goal-oriented motivation—and the presence of meaning in life. Similar findings were reported by Ahmad [42], who examined the relationships between academic motivation, learning styles and habits, and meaning in life. Both studies suggest that individuals pursuing specific goals may experience a stronger sense of meaning. Other research has further indicated that meaning in life may serve as both a predictor and a mediator of well-being through life adaptation [43,44]. Taken together, these findings imply that individuals with a strong sense of meaning in life may be more likely to report high levels of well-being. Based on these observations, the following hypotheses were formulated:

**H1.**  *Approach motivations are positively correlated with meaning in life and well-being.*

**H2.**  *Avoidance motivations are negatively correlated with meaning in life and well-being.*

A study conducted among Chinese adolescents [45] revealed an indirect association between mastery motivation and well-being, mediated by social comparisons and self-esteem. This suggests that the association between mastery motivation and well-being may depend on additional psychological factors. In a study with university students, Chen [46] found that mastery-approach and performance-approach goals were positively associated with life satisfaction, whereas performance-avoidance goals were negatively related to positive affect. These findings support the assumption that approach-oriented motivations are positively linked to well-being. Accordingly, the following hypothesis was tested:

**H3.**  *Meaning in life mediates the relationship between approach motivations and well-being.*

Romanian research on the motivations, preferences, and behaviors of MMORPG players [47] also highlighted achievement-related factors, including approach motivation, the sense of meaning derived from the activity, and positive emotional experiences. These results suggest that among players, approach motivations may be critical in sustaining engagement, especially when reinforced by a sense of meaning in gameplay. Thus, the final hypothesis was formulated:

**H4.**  *Gamers with approach motivations report higher levels of meaning in life and well-being than gamers with avoidance motivations.*

In summary, prior studies indicate that achievement motivation—particularly approach-oriented goals—contributes to a stronger sense of meaning in life, which in turn is associated with higher psychological well-being [43,48]. Moreover, meaning in life has been shown to mediate motivation and well-being, enhancing resilience and supporting adaptation [25,26]. Thus, meaning in life may constitute a key mechanism linking achievement motivation to well-being. However, relatively few studies have examined these relationships in the context of video gaming, even though the gaming environment—through competition, goal pursuit, and the experience of flow—represents a particularly relevant setting for investigating these dynamics [20,21]. The present study aims to address this gap by testing the mediating role of meaning in life in the relationship between achievement motivation and well-being among gamers.

It is important to emphasize that video game players represent a unique ecological context for studying achievement motivation, differing significantly from the general population in terms of goal structure. Video games are specifically designed to provide high-density feedback loops and optimal challenges, which are fundamental to the experience of ‘flow’ and the satisfaction of the basic psychological need for competence [18,49]. Unlike many spheres of daily life where goals are often ambiguous and feedback is delayed, video games offer environments where progression systems, such as experience levels, leaderboards, and skill trees, ensure that the correlation between effort and achievement is immediate, transparent, and measurable [50,51].

This specific architecture of gaming may intensify the relationship between motivation and meaning; within the micro-scale of a gaming session, a player experiences cycles of planning, execution, and reward that might take years to manifest in ‘real-world’ settings [52]. Studying this group thus allows for the observation of motivational mechanisms under conditions of exceptional goal clarity, making these findings a relevant benchmark for understanding how structural support for autonomy and competence in digital environments translates into broader existential resources and psychological resilience [18,53].

Based on the theoretical and empirical assumptions mentioned, the primary aim of this study was to examine the associations between achievement motivation and the other variables under investigation (H1–H3), as well as to compare approach- and avoidance-oriented types of achievement motivation in terms of levels of meaning in life and well-being among video game players (H4). The findings may apply to the video game industry by highlighting key characteristics of players, and, for similar reasons, may also have practical implications for educational and psychotherapeutic contexts.

## 2. Materials and Methods

### 2.1. Participants and Procedure

The study employed a cross-sectional design and was conducted in July 2025 using an online survey created with Google Forms. Participants were recruited through purposive sampling via Facebook groups dedicated to video gaming. Snowball sampling was also encouraged, with participants asked to share the survey with fellow university students who played video games. The study introduction provided information about anonymity and the estimated time required to complete the survey (approximately 15 min). All participants provided informed and voluntary consent to participate. The research protocol was approved by the Bioethics Committee of the Institute of Psychology at the University of Szczecin (KB 43/2025).

The study included 296 participants (Table 1), comprising 192 men (64.9%) and 104 women (35.1%), aged 18 to 35 years (*M* = 22.62, *SD* = 2.64). All participants were university students who played video games.

For group comparisons, participants were also categorized into dominant approach- and avoidance-motivation groups for exploratory comparative purposes. We acknowledge that this categorization is a simplification of the motivational continuum. However, it was employed to provide a clearer practical interpretation of the most prevalent motivational profiles within the gaming community. The approach-motivation group included 142 participants (98 men [69%], 44 women [31%]), aged 18 to 35 years (*M* = 23.09, *SD* = 2.79). The avoidance-motivation group included 154 participants (94 men [61%], 60 women [39%]), aged 18 to 35 years (*M* = 22.21, *SD* = 2.44).

### 2.2. Demographics

A custom sociodemographic questionnaire was also administered to collect data on age, gender, place of residence, and educational level.

### 2.3. Achievement Motivation

Achievement motivation was assessed using the Achievement Goals Questionnaire [54] (KCO), adapted from the Achievement Goal Questionnaire developed by Elliot and McGregor [24]. The instrument consists of 20 items rated on a 7-point Likert scale (1 = strongly disagree, 7 = strongly agree). The KCO measures four types of achievement goals. Two concern mastery goals, which focus on absolute standards (e.g., task requirements) or intrapersonal standards (e.g., past achievements or maximal personal capacities). Depending on goal orientation, individuals may evaluate these standards either positively (mastery-approach; 6 items) or negatively (mastery-avoidance; 5 items). The other two concern performance goals, which rely on interpersonal comparisons of achievement. Individuals may evaluate these comparisons positively (performance-approach; 5 items) or negatively (performance-avoidance; 4 items). Reliability analyses yielded a Cronbach’s α of 0.89 for the total scale. Subscale reliabilities were mastery-approach (0.81), performance-approach (0.85), mastery-avoidance (0.85), and performance-avoidance (0.80).

### 2.4. Meaning in Life

The Meaning in Life Questionnaire [25] (MLQ) was used to assess meaning in life. The Polish adaptation [55] consists of 10 items rated on a 7-point Likert scale (1 = absolutely untrue, 7 = absolutely true). The MLQ provides a total score as well as scores on two subscales: Presence of Meaning (the extent to which individuals perceive their lives as meaningful, significant, and valuable) and Search for Meaning (the active pursuit and engagement in finding meaning in life). Scores were calculated by summing up the items, with a potential range of 5 to 35 for each subscale. Higher scores reflect a higher presence of or search for meaning in life. Internal consistency was satisfactory, with Cronbach’s α = 0.78 for the total scale; subscale reliabilities were 0.89 for Presence of Meaning and 0.80 for Search for Meaning.

### 2.5. Well-Being

Well-being was measured with the WHO-5 Well-Being Index [56]. The Polish version [57] includes 5 items rated on a 6-point scale (0 = at no time, 5 = all of the time). Participants were asked to indicate how often they had experienced the described feelings or situations during the past two weeks. A higher score indicates a higher level of well-being. Reliability was satisfactory, with Cronbach’s α = 0.84.

### 2.6. Statistical Analysis

Statistical analyses were conducted using *IBM SPSS Statistics* (version 27.0). To test the mediation hypothesis, Model 4 of Hayes’ PROCESS macro [58] (version 5.0) was utilized. Preliminary data screening, involving boxplots and Mahalanobis distance, confirmed the absence of missing values and multivariate outliers. Pearson’s correlation coefficients were calculated to assess relationships between quantitative variables [59]. All estimates relied on 5000 bootstrap samples and 95% confidence intervals.

To enable valid comparisons across motivational orientations measured with a different number of items, raw subscale scores for approach- and avoidance-related achievement goals were first standardized within the full sample using z-score transformation. This procedure placed all motivational indicators on a common metric (*M* = 0, *SD* = 1), eliminating scale-length-driven distributional differences. Standardized scores were subsequently used to assign participants to dominant motivational-orientation subgroups for comparative analyses. Group membership was therefore based exclusively on standardized motivational scores, ensuring that the categorization reflected relative motivational dominance on the approach–avoidance continuum rather than absolute raw-score magnitude.

## 3. Results

### 3.1. Basic Descriptive Characteristics

The descriptive statistics, including means, standard deviations, and observed ranges for all study variables, are summarized in Table 2. Composite scores for each variable were calculated by summing the individual item responses for each respective subscale.

Regarding achievement motivation, participants reported the highest mean scores for mastery-approach goals (*M* = 30.11, *SD* = 6.71), followed by mastery-avoidance (*M* = 21.30, *SD* = 7.39) and performance-avoidance goals (*M* = 13.44, *SD* = 5.82). Performance-approach goals yielded the lowest mean scores in the sample (*M* = 16.25, *SD* = 6.87). In terms of meaning in life, scores for the search for meaning subscale (*M* = 24.51, *SD* = 6.31) were slightly higher than those for the presence of meaning (*M* = 21.19, *SD* = 8.02). Although the Shapiro–Wilk tests showed statistically significant (*p* < 0.05) deviations from normality for the remaining variables, their skewness and kurtosis values fell within the ±1 range. Preliminary normality checks (skewness and kurtosis) indicated that the data were appropriately distributed for the intended parametric analyses [60].

### 3.2. Correlation of Achievement Motivation, Meaning in Life, and Well-Being

Pearson’s correlation coefficients were calculated to examine the associations between the selected psychological variables (H1, H2).

As shown in Table 3, mastery-approach motivation was positively and significantly correlated with meaning in life (*r* = 0.19, *p* < 0.001), search for meaning (*r* = 0.15, *p* < 0.01), and presence of meaning (*r* = 0.13, *p* < 0.05). Performance-approach motivation was also positively correlated with meaning in life (*r* = 0.13, *p* < 0.05) and search for meaning (*r* = 0.12, *p* < 0.05). Conversely, mastery-avoidance motivation was negatively correlated with well-being (*r* = −0.41, *p* < 0.001) and presence of meaning (*r* = −0.30, *p* < 0.001), while positively correlated with search for meaning (*r* = 0.25, *p* < 0.001). Performance-avoidance motivation was negatively correlated with well-being (*r* = −0.19, *p* < 0.001) and presence of meaning (*r* = −0.13, *p* < 0.05), and positively correlated with search for meaning (*r* = 0.13, *p* < 0.05). Meaning in life demonstrated a moderate positive correlation with well-being (*r* = 0.44, *p* < 0.001), with the presence of meaning also showing a moderate correlation (*r* = 0.55, *p* < 0.001).

### 3.3. Mediation Analyses

**Figure 1:**
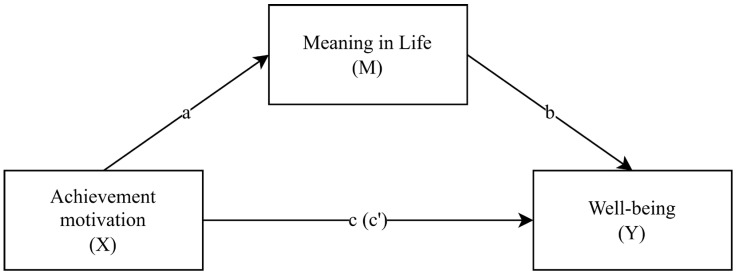
Diagram of the Analyzed Mediation Model.

To test the hypothesized mediating role of meaning in life, mediation analyses were conducted (Figure 1). Paths a, b, and c represent standardized regression coefficients and direct effects between two variables separately, while c’ represents the direct effect after controlling for the mediator. The results of the analyses for both mediation models are presented in Table 4.

The model examining mastery-approach motivation, meaning in life, and well-being demonstrated good fit to the data (*F*(2, 293) = 38.06, *p* < 0.001) and accounted for approximately 45% of the variance in well-being (*R*^2^ = 0.45). Initial regression between the independent and dependent variables (β = −0.02, *p* > 0.05) increased and became statistically significant (β = −0.08, *p* < 0.05) when meaning in life was included as a mediator. Standardized regression coefficients between the independent variable and the mediator (β = 0.30, *p* < 0.001) and between the mediator and the dependent variable (β = 0.22, *p* < 0.001) supported the proposed mediation model. The indirect effect size test confirmed a significant suppression effect (*Ind* = 0.07, 95% CI [0.02, 0.12]), indicating that meaning in life plays a significant mediating role between mastery-approach motivation and well-being, revealing an otherwise hidden relationship.

The second model examining performance-approach motivation, meaning in life, and well-being also demonstrated good fit to the data (*F*(2, 293) = 35.22, *p* < 0.001) and accounted for approximately 44% of the variance in well-being (*R*^2^ = 0.44). However, the analysis of the indirect effect did not support the mediation hypothesis. Although the standardized regression coefficient between the independent variable and the mediator was significant (β = 0.20; *p* < 0.05), the path from the mediator to the dependent variable was insignificant (β = 0.01; *p* > 0.05). Crucially, the bootstrap confidence interval for the indirect effect included zero (*Ind* = 0.04; 95% CI [−0.01, 0.09]), indicating that the mediation effect of meaning in life in the relationship between performance-approach motivation and well-being is not statistically significant.

### 3.4. Approach- and Avoidance-Motivation Group Comparisons

Independent-samples *t*-tests were conducted to examine differences between approach- and avoidance-motivation subgroups on selected psychological variables.

Results (Table 5) indicated that players with approach motivations reported significantly higher scores on overall meaning in life (*t*(294) = 4.44, *p* < 0.001; *M* = 48.42, *SD* = 10.92), presence of meaning (*t*(294) = 5.74, *p* < 0.001; *M* = 23.84, *SD* = 7.80), and well-being (*t*(294) = 5.52, *p* < 0.001; *M* = 14.59, *SD* = 4.57) compared to the avoidance-motivation subgroup (*M*_MLQ_ = 43.20, *SD* = 9.30; *M*_presence_ = 18.75, SD = 7.44; *M*_well-being_ = 11.57, *SD* = 4.84). Hedges’ g values indicated medium effect sizes (*g*_MLQ_ = 0.52; *g*_presence_ = 0.67; *g*_well-being_ = 0.64). No significant differences were found between groups in search for meaning (*t*(294) = 0.18, *p* > 0.05).

## 4. Discussion

The present study aimed to examine the relationship between achievement motivation, meaning in life, and well-being among gamers, as well as to investigate differences between approach- and avoidance-oriented motivation types. Overall, the statistical analyses largely supported the formulated hypotheses.

The type of achievement motivation was partially associated with meaning in life. Approach-oriented motivations were significantly positively correlated with meaning in life. Ahmad [42] reported similar findings in a study of university students, where academic achievement motivation positively correlated with meaning in life. These results suggest that striving for achievement may facilitate the construction of a meaningful life. No significant correlations were found between avoidance-oriented motivation and meaning in life, indicating that merely avoiding failure neither enhances nor diminishes one’s sense of life purpose. This may be explained by the fact that preventing failure does not generate positive experiences or support the pursuit of growth-oriented goals, which are critical for developing a sense of meaning [61]. Consequently, avoidance motivation may result in stagnation of meaning rather than regression.

The relationship between motivation type and well-being was supported only for avoidance motivations. The lack of significant correlations between approach-oriented motivations and well-being suggests that goal-directed striving does not necessarily translate into improved well-being. Research by Hygen et al. [62] found that social and novelty motivations in gamers were positively related to well-being, suggesting that approach motivations may sometimes be accompanied by goal-related pressure that limits well-being. Avoidance motivations, on the other hand, were negatively associated with well-being, consistent with prior research indicating that avoidance-oriented gamers may experience lower autonomy and increased external pressure, which significantly diminishes subjective well-being [55]. These findings imply that gamers with avoidance motivations may derive fewer positive experiences from gaming compared to their approach-motivation counterparts.

The analyses partially supported the mediating role of meaning in life in the relationship between approach-oriented motivations and well-being. For mastery-approach motivation, a suppression effect was observed: the direct association with well-being was negative, whereas the indirect path through meaning in life was positive. This negative direct relationship may suggest a ‘psychological cost of commitment,’ where mastery-oriented engagement in demanding gaming environments involves intense effort and performance pressure that may temporarily tax cognitive resources and reduce immediate subjective well-being. However, this same pursuit of excellence serves as a fundamental pillar for ‘life crafting’ and the development of agency. In this context, meaning in life acts as a critical psychological resource that ‘captures’ and transforms the strain associated with effort into a foundation for eudaimonic well-being. This suggests that while the pursuit of mastery can be inherently stressful, it becomes a source of happiness when players perceive their progress as part of a broader, meaningful life narrative [49]. Consequently, purposeful striving for mastery generates a dual effect: it creates a certain level of pressure (the observed negative direct path), while simultaneously reinforcing a sense of existential meaning. This meaning buffers the potentially negative side effects of effort, effectively transforming achievement motivation into a positive predictor of overall well-being [63].

In contrast, the mediation hypothesis for performance-approach motivation was not supported. Although striving to demonstrate competence was weakly associated with meaning in life, this relationship was insufficient to generate a significant indirect effect on well-being. This finding aligns with theoretical perspectives suggesting that performance goals, which rely on social comparison and external validation, may not foster the deep sense of personal significance required to sustain eudaimonic well-being to the same extent as mastery goals [34,55]. While mastery-approach motivation facilitates engagement and personal growth—key drivers of meaning [64]—performance-approach motivation appears to be a less stable predictor of well-being outcomes in the gaming context.

The group comparison hypotheses were also supported. Gamers with approach-oriented motivations reported higher levels of both meaning in life and well-being than those with avoidance-oriented motivations. These findings align with Csikszentmihalyi’s [49] concept of flow, which posits that engagement in activities that advance mastery and skill development facilitates the experience of flow, closely linked to meaning and psychological well-being. In this context, avoidance motivations may hinder the attainment of flow. However, Wang and Cheng [63] note that achievement motivation in gamers can be associated with problematic gaming behaviors, suggesting that in non-gaming populations, indicators of motivation and well-being might be substantially higher.

## 5. Limitations and Future Directions

Despite the obtained results, the present study has several limitations that should be considered when interpreting the findings. First, the cross-sectional nature of the research design precludes the establishment of definitive causal relationships between achievement motivation, meaning in life, and well-being. While mediation models were grounded in theoretical frameworks, the observed associations remain correlational. Thus, it is impossible to determine whether specific motivations lead to a greater sense of meaning or whether individuals with higher well-being are more predisposed to adopt approach-oriented goals. Future research should employ longitudinal or experimental designs to better elucidate the directional and mechanistic pathways underlying these dynamics.

Second, the definition of the study population was relatively broad, relying on self-reported identification as a “gamer” without accounting for specific behavioral characteristics. Data were collected via an online survey distributed through Facebook groups related to computer gaming. Consequently, the sample included only individuals who were members of these specific groups, thereby reducing its representativeness due to the survey’s limited reach. The sample was heterogeneous, yet it lacked detailed data regarding gaming frequency, session duration, or preferred genres (e.g., MMORPGs vs. competitive shooters). In future research, these variables should be included as potential confounding variables, as the intensity of engagement in gaming may moderate the relationships between achievement motivation and meaning in life [59]. This lack of granular control may limit the ecological validity of the findings, as different gaming contexts provide varying opportunities for mastery and social connection, which are known to influence psychological outcomes [62,63].

Third, the use of the student version of the Achievement Goal Questionnaire restricted the sample to individuals with at least a secondary education and aged 18 to 35 years, thereby limiting generalizability to the broader population. Online surveys are also susceptible to response biases. As noted by Couper and Zhang [65], respondents may provide socially desirable answers that do not reflect their true beliefs, fail to carefully attend to the survey items, or occasionally offer inaccurate information. Self-report measures are inherently subjective and may be linked by participants’ self-perceptions, potentially introducing systematic bias.

Fourth, a methodological limitation involves categorizing participants into approach- and avoidance-oriented groups based on their dominant scores. As noted in the methodological literature [66], the dichotomization of continuous variables can lead to a loss of information, reduced statistical power, and the potential artificial amplification of group differences. While this approach allowed for a simplified comparison of motivational profiles, it may not fully capture the complexity of individuals who exhibit high levels of both types of motivation. Future research should consider person-centered approaches, such as Latent Profile Analysis [67] (LPA), which can identify naturally occurring clusters of motivation without the inherent biases of manual categorization.

Furthermore, while a significant suppression effect was observed for mastery-approach motivation, its psychological underpinnings require further theoretical elaboration. The negative direct path to well-being, when accounting for meaning in life, suggests that the pursuit of mastery may entail a degree of strain or pressure that is only beneficial when translated into a sense of purpose. Future investigations should explore whether this “cost of mastery” is unique to the gaming context or reflects broader dynamics of “life crafting” in digital environments [64]. Incorporating objective metrics, such as in-game telemetry or time-logs, would also provide a more robust basis for assessing how gaming engagement interacts with personality traits to shape well-being.

Future research could expand on this study by incorporating instruments that address problematic gaming behaviors and by including comparison groups of non-gamers. This would allow verification of the relationships among approach-oriented motivation, meaning in life, and well-being beyond gaming populations, helping to determine whether the positive effects of approach motivation or other factors, such as behavioral dysregulation, drive observed group differences.

## 6. Conclusions

The findings of this study have potential implications for the computer gaming industry. Approach-oriented motivations were found to support a sense of meaning in life, thereby indirectly influencing gamers’ well-being. Games could be designed to promote gradual skill development, goal achievement, and exploration, thereby enhancing meaningfulness and facilitating flow experiences through sustained engagement. Features such as moral decision-making or allowing choices that are related to the game world may further deepen immersion and the sense of purpose.

The results concerning avoidance-oriented motivation suggest that games emphasizing failure or punitive mechanics may be less satisfying, as they can generate tension and anxiety associated with fear of making mistakes. Applying this knowledge to game design could enhance positive user experiences.

In educational contexts, the findings also have practical relevance. Educational programs structured around achievable, measurable goals and visible progress may increase motivation and reinforce the meaningfulness of learning activities. In contrast, approaches emphasizing fear of failure, which may reinforce avoidance motivation, should be replaced with formative teaching strategies that provide constructive feedback and support learning throughout the process, rather than solely relying on summative assessment and grading.

Given the growing popularity of computer games, particularly among younger populations, the results also have implications for psychotherapy. Understanding a player’s dominant motivation type can provide insight into the role of gaming in their life. For individuals with avoidance motivations, gaming may function as an escape from stress or fear of failure. Therapeutic work can therefore focus on exploring the client’s values, interests, goals, and aspirations to transform potentially maladaptive gaming patterns into behaviors aligned with the client’s personality and long-term well-being.

## Figures and Tables

**Table 1:** Demographic Characteristics of the Study Sample and Compared Subgroups (*N* = 296).

Variable	Research Sample (*N* = 296)		Players with Approach Motivation (*n* = 142)		Players with Avoidance Motivation (*n* = 154)	
	n	%	n	%	n	%
Sex						
Men	192	64.9	98	69.0	94	61.0
Women	104	35.1	44	31.0	60	39.0
Education level						
Vocational	6	2.0	2	1.4	4	2.6
Secondary	207	69.9	88	62.0	119	77.3
Higher	83	28.0	52	36.6	31	20.1
Size of Place of Residence						
Rural area	61	20.6	27	19.0	34	22.1
Town up to 50,000 inhabitants	41	13.9	18	12.7	23	14.9
Town from 50,000 to 100,000 inhabitants	38	12.8	18	12.7	20	13.0
City from 100,000 to 500,000 inhabitants	86	29.1	45	31.7	41	26.6
City over 500,000 inhabitants	70	23.6	34	23.9	36	23.4

**Table 2:** Descriptive statistics and results of the Shapiro–Wilk normality test (N = 296).

Variable	Min.	Max.	M	SD	Skewness	Kurtosis	W
Mastery-Approach Goals	6	42	30.11	6.71	−0.78	0.79	0.96 ***
Performance-Approach Goals	5	35	16.25	6.87	0.37	−0.32	0.98 ***
Mastery-Avoidance Goals	5	35	21.30	7.39	−0.23	−0.82	0.97 ***
Performance-Avoidance Goals	4	28	13.44	5.82	0.38	−0.49	0.97 ***
Meaning in Life	10	70	45.70	10.42	−0.40	0.75	0.98 **
Presence of Meaning in Life	5	35	21.19	8.02	−0.10	−0.87	0.97 ***
Search for Meaning in Life	5	35	24.51	6.31	−0.86	0.84	0.95 ***
Well-being	1	25	13.02	4.94	0.04	−0.60	0.99 *

W—Shapiro–Wilk statistics; * *p* < 0.05; ** *p* < 0.01; *** *p* < 0.001.

**Table 3:** Results of Pearson’s r Correlation Analysis among Selected Psychological Variables (*N* = 296).

	1	2	3	4	5	6	7
M-AP (1)	-						
P-AP (2)	0.35 *** [0.25, 0.45]	-					
M-AV (3)	0.41 *** [0.32, 0.50]	0.23 *** [0.12, 0.33]	-				
P-AV (4)	0.33 *** [0.22, 0.43]	0.56 *** [0.47, 0.63]	0.57 *** [0.48, 0.64]	-			
MLQ (5)	0.19 *** [0.08, 0.30]	0.13 * [0.02, 0.24]	−0.08 [−0.19, 0.04]	−0.02 [−0.14, 0.09]	-		
MLQ-P (6)	0.13 * [0.02, 0.24]	0.08 [−0.04, 0.19]	−0.30 *** [−0.40, −0.19]	−0.13 * [−0.24, −0.02]	0.80 *** [0.75, 0.84]	-	
MLQ-S (7)	0.15 ** [0.04, 0.26]	0.12 * [0.01, 0.23]	0.25 *** [0.14, 0.35]	0.13 * [0.02, 0.24]	0.64 *** [0.57, 0.70]	0.05 [−0.07, 0.16]	-
WB	−0.02 [−0.14, 0.09]	0.06 [−0.06, 0.17]	−0.41 *** [−0.50, −0.31]	−0.19 *** [−0.30, −0.08]	0.44 *** [0.34, 0.53]	0.55 *** [0.47, 0.63]	0.02 [−0.09, 0.14]

M-AP—mastery-approach goals; P-AP—performance-approach goals; M-AV—mastery-avoidance goals; P-AV—performance-avoidance goals; MLQ—meaning in life; MLQ-P—presence of meaning in life; MLQ-S—search for meaning in life; * *p* < 0.05; ** *p* < 0.01; *** *p* < 0.001.

**Table 4:** Results of Mediation Effect Analyses (Standardized Coefficients) (*N* = 296).

Model	a	b	c	c’	Indirect	95% CI	
						LL	UL
Mastery-Approach Goal Model	0.30 *** [0.12, 0.47]	0.22 *** [0.17, 0.27]	−0.02 [−0.10, 0.07]	−0.08 * [−0.16, −0.01]	0.07	0.02	0.12
Performance-Approach Goal Model	0.20 * [0.02, 0.38]	0.01 [−0.07, 0.26]	0.04 [−0.04, 0.16]	0.21 *** [0.16, 0.26]	0.04	−0.01	0.09

Mastery-Approach Goal Model—mediating effect of meaning in life between mastery-approach motivation and well-being; Performance-Approach Goal Model—mediating effect of meaning in life between performance-approach motivation and well-being; LL—lower level of 95% confidence interval; UL—upper level of 95% confidence interval; * *p* < 0.05; *** *p* < 0.001.

**Table 5:** Results of Independent-Samples t-Test (*N* = 296).

Variable	Approach (n = 142)		Avoidance (n = 154)		F	t	g
	M	SD	M	SD			
MLQ	48.42	10.92	43.20	9.30	3.50	4.44 ***	0.52
MLQ-P	23.84	7.80	18.75	7.44	0.36	5.74 ***	0.67
MLQ-S	24.58	6.53	24.45	6.12	0.98	0.18	0.02
WHO-5	14.59	4.57	11.57	4.84	0.32	5.52 ***	0.64

Approach—Players with approach motivation; Avoidance—Players with avoidance motivation; F—Brown-Forsythe test; g—Hedges’ g; MLQ—meaning in life; MLQ-P—presence of meaning in life; MLQ-S—search for meaning in life; WHO-5—well-being; *** *p* < 0.001.

## Data Availability

The dataset analyzed during the present study is available in the OSF repository and can be accessed at https://osf.io/9zevt/files/osfstorage/6868533115c21bcf9549f2fe (accessed on 8 January 2026).
